# Work–Life Balance: Can You Actually Make That Happen?

**DOI:** 10.3389/fped.2015.00117

**Published:** 2016-01-06

**Authors:** Dahui You

**Affiliations:** ^1^Department of Pediatrics, University of Tennessee Health Sciences Center, Memphis, TN, USA

**Keywords:** biomedical research, working mother, efficiency, work–life balance, stress

Let me start off by saying – there is no such thing as “work–life balance” in a biomedical research career. In today’s modern society, the world is evolving at an unprecedented fast pace. The speed of progress in the biomedical research field is astonishing. The publications indexed by PubMed was ~9,000 during 2000 and increased to ~60,000 during 2013 (1). Unfortunately, the growth of federal and private funding for biomedical research is much slower. In fact, it is estimated that the resources that National Health Institutes retains today are less than 75% than they were in 2003 (2). The ever-increasing competition for funding makes work–life balance almost impossible. From a personal perspective, I have not yet encountered a successful scientist with a laid-back lifestyle throughout my research career from 2004 (when I became a graduate student) to present day. In fact, in my experience, most successful scientists struggle with busy schedules and lack of work–life balance. My Ph.D. mentor – Dr. Stephania Cormier – who is a tenured, full professor now, started her career as an assistant professor in 2002. She is highly intelligent and disciplined to a level that intimidates people; however, she still constantly works during weekends and holidays to clear off her work lists. When I was a student or postdoctoral trainee, I dedicated my life to research. Some of my experiments were inherently time-consuming, so I had to stay up till 03:00 a.m. the next day although I started at 07:00 a.m. At times that I returned home at regular hours, I read papers, books or tutorials related to my research. I did not have a life outside of my research, and at that time I did not feel that I needed one. Research was fun and the excitement from a successful experiment, publication, or presentation for a national meeting was satisfying… until my son Arthur was born 3 years ago. He is demanding and exhausting, but also amazingly adorable. Naturally, as a mother I want to spend time with him, and do not want to miss a single step that he makes as he grows up. This change in my life inevitably affected my dedication to my career. All the extra hours that I used to commit to research are gone; I suddenly need a life away from the bench. The only option I have is to achieve a work–life balance, even if it seems a “mission impossible.”

This may sound depressing, but realizing the truth is the first step to fix the problem. The problem is time! My workload has remained relatively the same, but now the time I have to complete tasks is about two-thirds of the time I had before having my son. This means that I must increase my efficiency at work one-and-a-half fold. To most scientists, including myself, who are already highly efficient, it is difficult to further increase efficiency. With this in mind, I have researched effective time management methods and practiced a few principles (3, 4). Here are some tips that have really helped me.

## Prioritize the Night Before

Every evening before I go to bed, I go through a list of tasks that needs to be completed, and plan my time for the next day. Some may prefer to prioritize tasks every morning, I have just found that I sleep better if I know exactly how the following day will look like and I wake up with a clear mind, focused on my priorities of the day. How to prioritize tasks is an art in itself, especially when there are so many things that seem equally important at the time. One key principle to keep in mind is to only schedule about two-thirds of the work time. For example, if I plan to work 8 h, then I only schedule 5-h workload. The reason behind this is that if I can focus on the priorities for 5 h, most of time I can finish them with high efficiency. The other 3 h is for unscheduled interruptions that happen every day. In order to schedule just enough workload, I need to have a fairly accurate estimation of the time of each task. I achieved this by logging my activity each day for about a half year and then averaging the time that I spent on each task. Another important principle is to start the day with the most difficult task so that the day gets easier when your energy level and focus gradually goes down.

## Do Not Multitask

Multitasking may help increase efficiency if the tasks are not very demanding (5). Unfortunately, most of the daily tasks that a scientist takes need focus, such as writing, reading, and troubleshooting experiments. Multitasking on these tasks will only distract you and decrease your efficiency (6). When I write grants or manuscripts, I make sure that I have dedicated writing time and I minimize distractions. After shutting the office door, turning off Outlook, and putting away my phone, I write for about 45 min. Then I try to answer emails and phone calls in 20 min and go back to writing for another 45 min and so on. In this way, I only focus on one thing at a time and it actually increases my efficiency.

## Get Help

I have a very shy personality and asking for help does not come naturally to me. However, I realized that I have to do this to be able to survive and be successful at my job. I ask for all kinds of help, work and non-work related. My mother stayed with us when Arthur was born. I “bother” my mentors with grants, manuscripts, teaching skills, and even my struggles with work–life balance. I submit my manuscripts/grants to internal editing before actual submission. Nevertheless, the most help I get is from graduate students and postdoctoral trainees. I cannot do the experiments all by myself; in fact, trainees in a typical laboratory setting perform most experiments. There is plenty of information out there about how to find a good student and a postdoctoral trainee. I personally do not have much experience in the interviewing and hiring process as a young scientist. However, my experience has shown me that finding a motivated and personality-matched trainee is the key in research where experimenting is the cornerstone. Besides getting help on performing experiments, I also ask trainees to help me review or write manuscripts/grants. This is a win–win situation. The trainees obtain adequate training on manuscripts/grants writing skills during the actual process of helping complete these tasks. This mentoring method has been demonstrated successfully elsewhere (4).

## Exercise

Everyone can see the benefits of exercising. It boosts your health and energy and, therefore, in the long run, it will increase work efficiency and quality of life in general. What I want to emphasize is that exercise is the perfect time for multitasking! I exercise regularly about four times a week, with a six- to eight-mile run during weekends. While exercising, I often think about the big questions in my life: is there anything that I am not happy about myself at work or in my personal life? How will I fix it? I also think about specific aspects of my work: why is my data not consistent? How do I probe my hypothesis by an experiment? What will be the next big wave of progress in my research field? In fact, I would say running is the most quality time I have for thinking. There is ample evidence suggesting that creativity happens in a moment that the brain is not highly focused nor distracted (7). This is called the drifting phase of the brain. When running, my mind wanders, takes its time, and eventually centers on the questions aforementioned. The benefit is enormous and running is not at all boring to me. My suggestion is to find a way to exercise regularly with no interruptions and just enjoy the Zen moment.

## Be Flexible and Prepared for Uncertainty

When I was a student and a postdoctoral trainee, I had a fairly fixed schedule due to my experiments. I worked with neonatal mice, and when the mice were born, I had to do the experiments on certain days, irrespective of weekends or not. When I did flow cytometry experiments, it was a 13-h procedure. I had limited control of my schedule. Now that most of my time is spent on writing grants, manuscripts, and reading, I find myself having the luxury of a more flexible schedule. I can work from home if Arthur is sick, or the daycare is closed. I can shuffle my tasks around so small tasks can fit into my schedule. However, everyone who writes knows that writing and thinking need continuous motion. The time from being interrupted to return to focus mode varies among people, but decreasing this time is the key for being efficient and flexible at work. I myself still struggle with it. I can hardly concentrate when Arthur is around and asking questions all the time. So while I can work from home when he is sick, my efficiency is low. The only trick that works for me is meditation and I am still not satisfied with my progress.

Being flexible also means to be prepared for uncertainty. I find myself, as many working moms, constantly dealing with chaos due to unexpected reasons related to my son. I unfortunately learned in a hard way that procrastination is not an option and completing tasks ahead of time is the only way to be prepared for uncertainty (3). One day, I was going to give a lecture in the evening. I had scheduled the whole day for preparation since it was my first lecture on that topic. However, my son’s daycare was unexpectedly closed and I had to prepare the lecture while my son was around. The efficiency was of course extremely low and I barely made my slides on time. The lecture turned out OK, but I wished that I had prepared the lecture much earlier. With uncertainty in mind, I now always try to finish important tasks ahead of time.

Finally, keep in mind that it is OK if your work–life balance is temporarily broken. Nobody can completely control life. And if so, what is the fun of life? So be open-minded, embrace the uncertainty, and accept the interruption. As a matter of fact, I stayed up late last night to write this article and I am still late for the deadline to my editor. “*C’est la vie*.” Live with it.

## Conflict of Interest Statement

The author declares that the research was conducted in the absence of any commercial or financial relationships that could be construed as a potential conflict of interest.
